# Core endophyte communities of different citrus varieties from citrus growing regions in China

**DOI:** 10.1038/s41598-020-60350-6

**Published:** 2020-02-27

**Authors:** Shahzad Munir, Yongmei Li, Pengfei He, Min Huang, Pengbo He, Pengjie He, Wenyan Cui, Yixin Wu, Yueqiu He

**Affiliations:** 1 State Key Laboratory for Conservation and Utilization of Bio-resources in Yunnan, Yunnan Agricultural University, Kunming, 650201 Yunnan China; 2 Agriculture College and Urban Modern Agriculture Engineering Research Center, Kunming University, Kunming, 650214 Yunnan China; 3 National and Local Joint Engineering Research Center for Screening and Application of Microbial Strains, Kunming, 650217 Yunnan China; 4 Faculty of Agronomy and Biotechnology, Yunnan Agricultural University, Kunming, 650201 Yunnan China

**Keywords:** Soil microbiology, Microbial ecology

## Abstract

The native microbiomes of citrus trees play important roles in plant health, with good communication between the native microbiome and the host plant. Here, we report on the native endophytes in 24 citrus varieties in nine citrus growing regions in China; some of the trees were healthy and others had asymptomatic or symptomatic huanglongbing, which is caused by the pathogen *Candidatus* Liberibacter asiaticus (*C*Las). We used culture-dependent analysis and characterized the isolates by partial 16S rRNA gene sequencing. The endophytes were compared between different citrus varieties, regions, and disease states (healthy, asymptomatic, and symptomatic). The total number of endophytes isolated from most of the citrus varieties was 10^4^–10^6^ CFU/g of leaves, but it differed significantly by disease state, with the highest numbers in the healthy leaves and the lowest in the symptomatic leaves (*p* < 0.05). Among the citrus varieties, the Valencia variety had the maximum number of endophyte species (22). The most dominant endophytes were *Bacillus subtilis*, *B. velezensis*, *Curtobacterium luteum*, and *Microbacterium testaceum*. The higher frequency of *B. subtilis* in the healthy/asymptomatic plants compared to the symptomatic plants suggests that it has a role in huanglongbing resistance. Native endophyte communities in various citrus varieties could be used to improve citrus growth and combat *C*Las.

## Introduction

Most plants are hosts to a diverse group of bacteria, known as endophytes, that do not harm the host and colonizing the internal tissues of plants without causing any immediate and overt negative symptoms^1^. Plant physical and physiological barriers need to be overcome for successful colonization by endophytes, with the exception of colonization by vertically transmitted endophytes. There is a clear distinction between pathogens and endophytes, as the latter do not harm or destroy plant cells to obtain resources. Putative pathogen effector proteins can act as important signatures of the divergence underlying host specialization^2–4^. However, there have been no significant molecular studies on the host specialization of endophytes that differentiate endophytes from pathogens^5^.

The rhizosphere contains rhizodeposits and root exudates, which are important residues for attracting microorganisms from the surrounding environment^6,7^. Bacterial endophytes mainly enter the host plant via colonization of root hairs^8^, and another important route involves attraction of endophytes by the exudates of leaf and stem surfaces^6^. Only adapted bacteria have the ability to enter plants through hydathodes, stomata, and wounds. Reductions in surface bacteria colonization can occur due to a lack of nutrients, ultraviolet light and, most importantly, desiccation^6,9^. Various bacterial endophytes with different colonization routes and specific bacteria–host interactions have been described in detail^9,10^. Several active and passive mechanisms are involved in the movement of endophytic bacteria from the rhizoplane to the cortical cell layer, and further colonization involves crossing the endodermis^6,11^. The internal plant compartments can be systematically colonized by bacterial endophytes using the xylem vascular system as the main route, and some bacterial endophytes colonize locally via intercellular spaces^10^. The holes in the perforation plates between xylem elements are large enough for endophytes to pass through^12^, but it may take several weeks for the vertical spread of bacteria through specific plants, and it remains unclear why this dissemination is so slow^13^. The optimal mechanisms by which specific endophytes reach specific parts or tissues of plants remains unknown.

Core microbial communities are responsible for specific functions within ecosystems^14,15^. Plant microbiomes comprise hundreds to thousands of operational taxonomic units (OTUs), with a small number of taxa representing a small proportion of the overall abundance of bacteria having dominant roles^16–18^. Both biotic factors such as plant developmental stage, phytopathogens and abiotic factors such as soil type, climate, and season can restructure the plant microbiota^19,20^. Much remains unknown about the core microbiome function and importance for plant health, as a limited number of studies have been conducted on the core microbial communities of specific plants^17,21,22^.

Plant disease development can negatively affect the plant microbiome^17,23,24^. Plant diseases induce complex changes in plant-associated microbial communities; for example, *Rhizoctonia solanacearum* infection of tomato plants causes drastic decreases in the dominant microbial communities in the rhizosphere^23^. Beetroot rot disease caused by various *Rhizoctonia* species can be suppressed by beneficial bacteria in beet plants, such as *Burkholderiaceae*, *Lactobacillaceae*, *Pseudomonadaceae*, and *Xanthomonadales*^25^. In contrast, synergistic interactions among plant pathogens can cause or enhance diseases such as broccoli head rot, tomato pith necrosis, and mulberry wilt^26^. Novel strategies involving native bacteria may help to combat plant pathogens; biocontrol bacteria isolated from plants have shown promising results in the lab^27–29^, but little success has been achieved under field conditions^30,31^. However, identifying beneficial native endophytes from various citrus varieties under pathogen stress could possibly be used to control citrus pathogens and other plant pathogens. Similar trends has been reported regarding the devastating citrus disease, huanglongbing (HLB), which is caused by *Candidatus* Liberibacter asiaticus (*C*Las), *Candidatus* Liberibacter americanus, and *Candidatus* Liberibacter africanus^32^, resulting in severe losses to the citrus industry worldwide^33^. It has previously been shown that the restructuring of the citrus microbiome caused by HLB disease development could be overcome using native bacteria to manage the pathogen titer^34,35^.

Using 16S rRNA gene, this study aimed to identify the native culturable endophytes in the leaves of healthy citrus varieties and citrus varieties with asymptomatic or symptomatic huanglongbing (caused by *C*Las) in nine citrus growing regions in China. Further, the differences in the core endophyte communities among different citrus varieties were compared. Moreover, the endophytes that were common among different citrus varieties were identified.

## Materials and Methods

### Plant samples, citrus varieties, and locations

**Figure 1:**
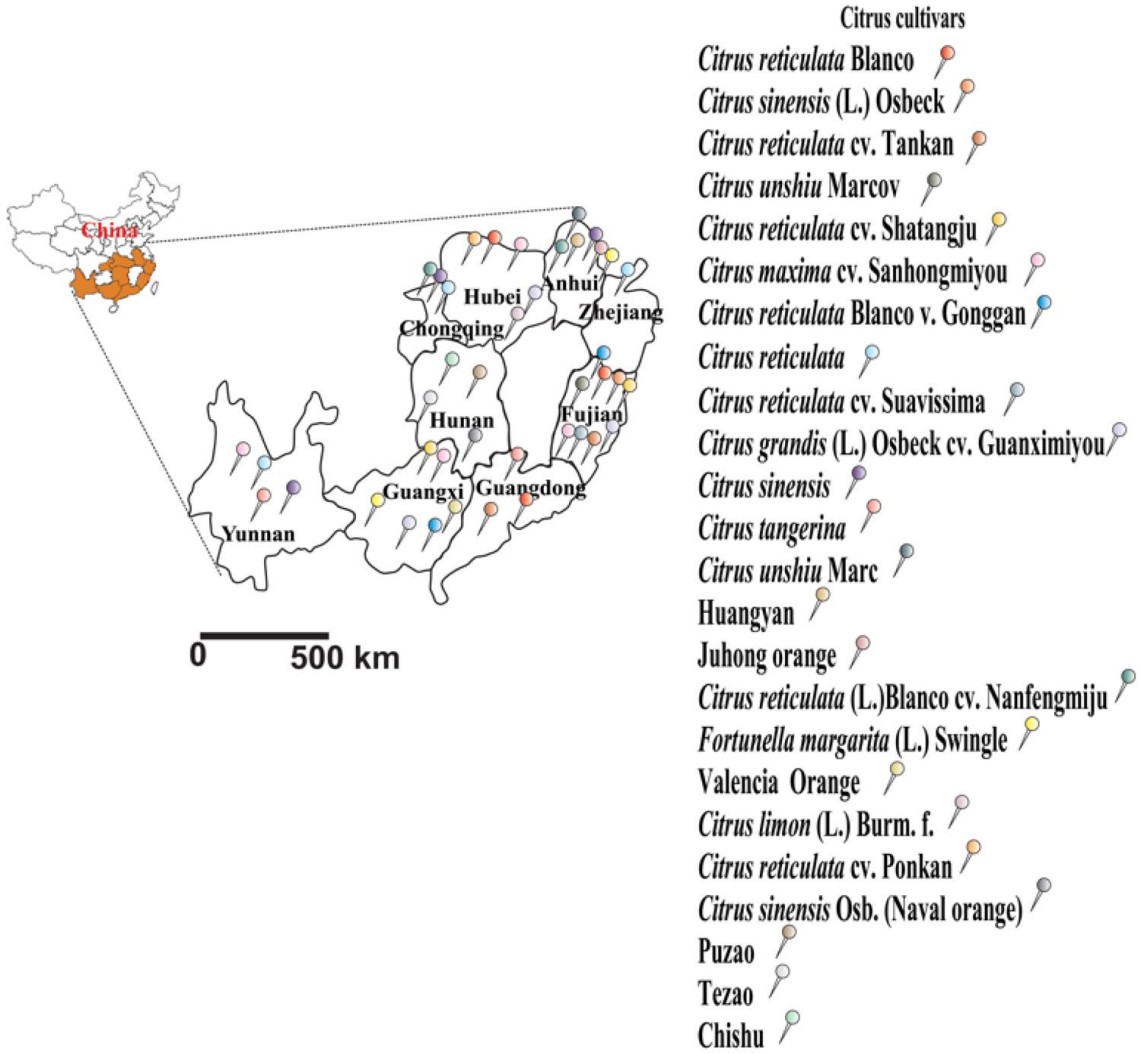
Map of nine citrus growing regions in China, with the 24 citrus varieties depicted with different symbols. Leaves included healthy, symptomatic, and asymptomatic leaves.

Citrus plants from citrus growing regions in nine provinces (Yunnan, Fujian, Anhui, Guangxi, Hubei, Hunnan, Guangdong, Chongqing, and Zhejiang provinces) in China (Fig. 1) were sampled in 2016–2018. The citrus varieties comprised *Citrus reticulata* Blanco*, C. sinensis* (L.) Osbeck, *C. reticulata* cv. Tankan*, C. unshiu* Marcov. forma Miyagawa-wase × *C. sinensis* (L.) Osbeck*, C. reticulata* cv. Shatangju*, C*. maxima cv. Sanhongmiyou*, C. reticulata* Blanco var. Gonggan*, C. reticulata, C. reticulata* cv. Suavissima*, C. grandis* (L.) Osbeck cv. Guanximiyou*, C. sinensis, C. tangerina*, *C. unshiu* Marc, Huangyan, Juhong orange*, C. reticulata* (L.) Blanco cv. Nanfengmiju*, Fortunella margarita* (L.) Swingle, Valencia orange*, C. limon* (L.) Burm. f., *C. reticulata* cv. Ponkan, and *C. sinensis* Osb. (navel orange) and, based on the Chinese names, Puzhao, Tezao, and Chishu. Three citrus trees (which were considered as a single sample pool) were randomly selected for each citrus variety in each citrus grove (with one grove per region). Nine leaves were collected from each tree across all ordinate directions. The leaves were divided into healthy leaves (with no physical appearance of symptoms), asymptomatic leaves (with no physical appearance of symptoms along with the following qPCR results: CT value: 25–32 [10^4^ *C*Las pathogen copies/g of leaves]), and symptomatic leaves (with HLB).

### Sample processing and DNA extraction for detection of *Candidatus* Liberibacter asiaticus (*C*Las) pathogen

Samples were subsequently processed for PCR template preparation. DNA was extracted using the cetyl trimethylammonium bromide (CTAB) method with slight modifications^36,37^. Briefly, the midribs of leaves were separated and frozen in liquid nitrogen. The midribs of the nine leaves from a single sample pool were then macerated with a sterile pestle and mortar. The resulting powder was transferred into sterile Eppendorf tubes, CTAB buffer (1 ml) was added, and the samples were incubated in a water bath (65 °C) for 30–60 min. Next, phenol: chloroform: isoamyl alcohol (1:1:5, v/v) was added to each tube, which was then centrifuged (12,000 rpm, 10 min). After transferring the supernatant into a fresh tube, a mixture of isopropanol and sodium acetate (3 M) (1:1 v/v) was added and centrifugation was performed again (12,000 rpm, 10 min). Ethanol (70%) was used to wash the resulting pellet. The centrifugation was performed one additional time to remove impurities from the DNA. The residual ethanol was allowed to evaporate, and sterile distilled water was used to resuspend the DNA pellet. The DNA was stored at −20 °C until further use.

### qPCR for detection of *C*Las pathogen

*C*Las was detected using SYBR Green I reagent (Bio-Rad). PCR was performed in a 25-µl reaction mixture containing DNA template (5 µl), 1 × PCR buffer (SYBR Green Master Mix; Bio-Rad), and 0.8 µm CQULA04R and CQULA04F primers (which amplify the *C*Las-specific sequence of the ribosomal protein L12 [rplL] gene)^38^. A StepOne Real-Time PCR System (Applied Biosystems) was used with the following program: 95 °C for 1 min and 45 cycles each of 95 °C for 15 s, 59 °C for 15 s, and 72 °C for 45 s. During the extension step (72 °C for 45 s) of each cycle, the instrument collected the fluorescent signal generated by SYBR Green I (Bio-Rad) nonspecifically bound to any dsDNA. To analyze the specificity of the PCR amplification, a melt curve analysis was subsequently conducted using the following program: 95 °C for 1 min, 55 °C for 1 min, and then the temperature was increased by 0.5 °C every 10 s from 55 °C to 95 °C. The melt curve was plotted according to the manufacturer’s instructions (Bio-Rad).

### Construction of recombinant plasmid and standard curve

A target fragment (382 bp) of *C*Las was amplified using the CQULA03F/CQULA03R primer set^38^ and electrophoresed on 2% agarose gel with UltraPower DNA stain (Bioteke Corporation). The target band was cut out and purified using a gel extraction kit (Omega) according to the manufacturer’s instructions. The DNA was eluted in 20 μl Millipore pure water and 7.5 μl DNA with 2.5 μl Solution 1 (Takara) was used for ligation with the pMD18-T vector (Takara). The recombinant plasmid (pUC18–382) solution (10 µl) was used to transform 50 µl competent *Escherichia coli* TG1 cells (Takara) for 8–10 h at 4 °C. The resulting mixture was spread onto Luria Bertani (LB) plates containing ampicillin and positive clones were confirmed by PCR using *C*Las-specific primers^37^. The positive recombinant plasmid was extracted using a HiBind DNA Mini Column (Omega), following the instructions for the Plasmid DNA Mini Kit (Omega). Enzyme digestion with 4 µl Q-Pst1 and electrophoresis on 1% agarose gel was performed to confirm the insertion in the plasmid (Fig. S1). The recombinant plasmid was then sequenced and aligned using BLASTn. The plasmid standard solution was quantified with a NanoDrop spectrophotometer (Ultrospec 2100 pro, Amersham Biosciences). The solution was then diluted using 10-fold serial dilutions (to 10^−10^) and the dilutions were subjected to RT-PCR to generate a standard curve (Fig. S2). Subsequently, a real-time thermal cycler could be used to automatically calculate the pathogen titer in the field samples. The unit of detection was fg µl^−1^, which was converted into pathogen copy number/g of leaves. A melt curve was plotted according to the manufacturer’s instructions (Bio-Rad) (Fig. S3).

### Isolation of citrus native endophytes

The native endophytes were isolated from the healthy, asymptomatic, and symptomatic citrus leaves. Briefly, the citrus leaves were washed three times with tap water and then surface sterilized as reported previously^36^ to avoid contamination of the analysis by surface bacteria. To confirm that the endophytes analyzed were native to the citrus leaves, a sterility check was performed by plating 100 μl of the water from the third rinse on Luria Bertani (LB)/Tryptic Soya agar medium. The leaves were then cut into four pieces (5–6 mm long), plated on LB/TSA) plates, and incubated for 48–96 h at 37 °C. Single colonies of bacteria recovered from each leaf fragment were selected, purified by repeated streaking, and stored in 50% glycerol in a −80 °C freezer. The bacterial endophytes were selected for further analysis by choosing all endophytes with unique morphology (based on colony shape and color). The total number of endophytes (CFU/g of leaves) for each citrus tree was also recorded. For each citrus variety, six plates each containing four leaf pieces were analyzed. Additionally, using citrus seedlings in a greenhouse, we confirmed that the endophytes could easily disseminate inside citrus leaves.

### Endophyte DNA extraction and PCR amplification

The bacteria were grown until mid- to late-log-phase (0.5–0.7 at OD_600_) and 1 ml of the culture was centrifuged at 7,500 rpm for 10 min. DNA was extracted using the CTAB method with slight modifications^36^. The pellet was resuspended in Tris-ethylenediaminetetraacetic acid (EDTA) buffer and 525 μl phenol: chloroform: isoamyl solution (25:24:1, v/v) was added to the tube followed by centrifugation at 12,000 rpm for 15 min. An equal volume of chilled isopropanol was added to the resulting supernatant and the solution was centrifuged again at 12,000 rpm for 15 min. The pellet was resuspended in 50 μl distilled water and then left overnight at 4 °C. The presence and concentration of bacterial DNA was confirmed by running 5 μl of product on a 1.5% agarose gel. Purified DNA appeared as a defined band when visualized under ultraviolet light.

Identification of the endophytes was performed by amplifying the 16S rRNA gene from the selected bacteria^39^ using IDB-PO 5′-GAAGAGTTTGATCCTGGCTCAG-3′ and 5′-IDB-P6 CTACGGCTACCTTGTTACGA-3′ primers^27^. The amplification conditions (repeated twice) were as follows: initial denaturation at 94 °C for 4.5 min followed by 30 cycles of denaturation at 94 °C for 40 s, annealing at 55 °C for 30 s, and extension at 72 °C for 1 min followed by a final extension at 72 °C for 10 min. The positive controls were the pathogenic bacteria of maize top rot, *Klebsiella pneumoniae* KpC4, and the clinical strain Kp138. Purified amplified products were cloned into the vector Top10 (Tiangen) for sequence analysis.

### Phylogenetic analysis

Phylogenetic trees were constructed to determine the taxonomic relationships using Molecular Evolutionary Genetics Analysis software (MEGA7.0.21) and the maximum likelihood method based on the Kimura two-parameter model^40^. The dominant endophytic sequences obtained in this study have been submitted to the GenBank database (accession numbers MK618592–MK618638).

### Statistical analysis

The data obtained from different provinces and different citrus varieties were subjected to analysis of variance (ANOVA) followed by Duncan’s multiple range test. *p* < 0.05 was considered statistically significant. SPSS v21 (IBM) was used for the statistical analysis.

## Results

### Endophytic diversity in citrus varieties in different provinces

**Figure 2:**
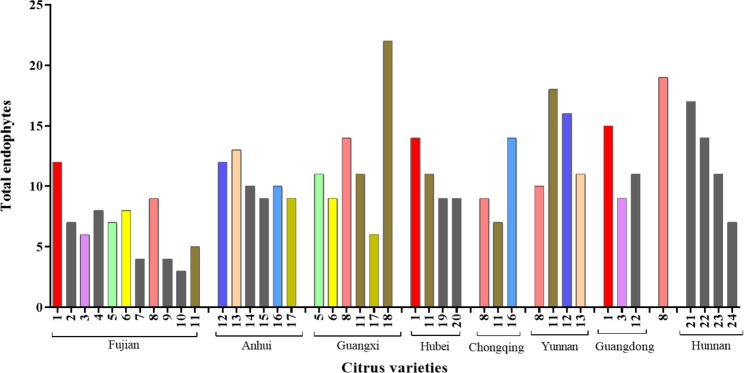
Total endophytic bacteria from 24 citrus varieties from nine citrus growing regions in China. Provinces with different citrus varieties have diverse endophytes. The bar representing only variety 8 corresponds to the Zhejiang province. The dark grey bars represent the non-repeated varieties in different provinces. Citrus varieties are labeled with the same numbers in different provinces: 1, *Citrus reticulata* Blanco; 2, *C. sinensis* (L.) Osbeck; 3, *C. reticulata* cv. Tankan; 4, *C. unshiu* Marcov. forma Miyagawa-wase × *C. sinensis* (L.) Osbeck; 5, *C. reticulata* cv. Shatangju; 6, *C. maxima* cv. Sanhongmiyou; 7, *C. reticulata* Blanco v. Gonggan; 8, *C. reticulata;* 9, *C. reticulata* cv. Suavissima; 10, *C. grandis* (L.) Osbeck cv. Guanximiyou; 11, *C. sinensis*; 12, *C. tangerine*; 13, *C. unshiu* Marc; 14, Huangyan; 15, Juhong orange; 16, *C. reticulate* (L.) Blanco cv. Nanfengmiju; 17, *Fortunella margarita* (L.) Swingle; 18, Valencia orange; 19, *C. limon* (L.) Burm. f.; 20, *C. reticulata* cv. Ponkan; 21, *C. sinensis* Osb. (navel orange); 22, Puzao; 23, Tezao; 24, Chishu.

The native bacterial endophytic communities of healthy, symptomatic, and asymptomatic citrus leaves in nine citrus growing regions was assessed in 2016–2018, and Fig. 2 show the endophytic microbial diversity of different citrus varieties from different citrus growing regions in China. The endophyte communities varied between specific varieties from different sites and between different varieties from the same site. Diverse endophytic bacteria were recovered from the same and different varieties located in multiple site or individual sites, respectively. Fujian province had a large range of citrus varieties, which resulted in the maximum endophyte isolation frequency, with *C. reticulata* Blanco having more endophytes in Fujian. However, the number of endophytes depends on the number of citrus varieties in each specific location; for example, the high endophyte isolation frequency in Fujian province was due to the collection of samples from 11 citrus varieties. Fewer endophytes were recovered from *C. grandis* (L.) Osbeck cv. Guanximiyou in Fujian province. Chongqing province had only 30 endophytes. Among all of the citrus varieties from different provinces, Valencia Orange had maximum endophyte species (22). In addition, citrus varieties in different and same provinces were different in terms of endophyte recovery. The total number of endophytes isolated from most of the citrus varieties was 10^4^–10^6^ CFU/g of leaves, with no significant differences between citrus varieties (Table S1).

### Dominant endophytes in citrus leaves

**Figure 3:**
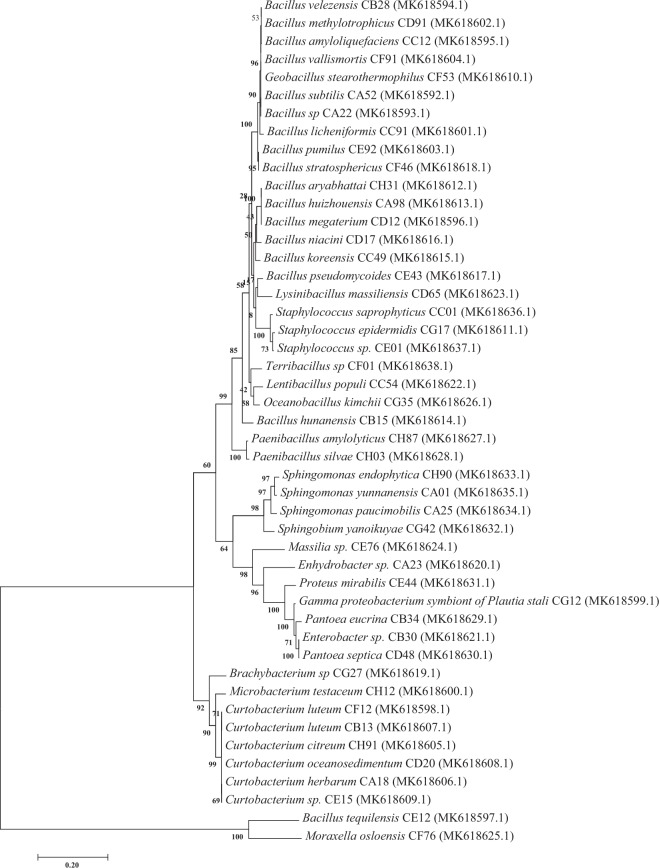
Phylogenetic tree of dominant endophytes based on the 16S rRNA gene. The evolutionary history was inferred using the Maximum Likelihood method based on the Kimura two-parameter model and the analysis involved 11 nucleotide sequences. The percentage of trees in which the associated taxa clustered together is shown next to the branches. All positions with gaps and missing data were eliminated. The analysis was conducted in MEGA7.

The dominant bacterial endophytes isolated from the various citrus varieties were *Bacillus subtilis, Bacillus* sp., *B. velezensis, B. amyloliquefaciens, B. megaterium, B. tequilensis, Curtobacterium luteum, Gammaproteobacterium symbiont of Plautia stali, Microbacterium testaceum, B. licheniformis, B. methylotrophicus, B. pumilus, B. vallismortis, Curtobacterium citreum, C. herbarum, C. luteum, C. oceanosedimentum, Curtobacterium sp., Geobacillus stearothermophilus, Staphylococcus epidermidis, B. aryabhattai, B. huizhouensis, B. hunanensis, B. koreensis, B. niacini, B. pseudomycoides, B. stratosphericus, Brachybacterium* sp., *C. oceanosedimentum, Enhydrobacter* sp., *Enterobacter* sp., *Lentibacillus populi, Lysinibacillus massiliensis, Massilia* sp., *Moraxella osloensis, Oceanobacillus kimchii, Paenibacillus amylolyticus, P. silvae, Pantoea eucrina, P. septica, Proteus mirabilis, Sphingobium yanoikuyae, S. endophytica, S. paucimobilis, S. yunnanensis, S. saprophyticus, Staphylococcus* sp., and *Terribacillus* sp. (Table 1). Figure S4 shows how pure cultures of dominant endophytes were obtained by streaking individual colonies on LB agar. Phylogenetic analyses of the dominant isolates are shown in Fig. 3.

**Table 1:** Total isolation frequency of dominant native bacterial endophytes isolated from different citrus varieties from different citrus growing regions in China.

Strain ID	Organism	Isolation source	Region	Accession No.	Seq. identity%
CA52	*B. subtilis*	Citrus tree	China	MK618592	100
L1–21	*B. subtilis*	Citrus tree	China	*CGMCC15726	99
CA22	*Bacillus* sp.	Citrus tree	China	MK618593	100
CB28	*B. velezensis*	Citrus tree	China	MK618594	99.93
CC12	*B. amyloliquefaciens*	Citrus tree	China	MK618595	99.93
CD12	*B. megaterium*	Citrus tree	China	MK618596	100
CE12	*B. tequilensis*	Citrus tree	China	MK618597	99.79
CF12	*Curtobacterium luteum*	Citrus tree	China	MK618598	99.93
CG12	*Gamma proteobacterium symbiont of Plautia stali*	Citrus tree	China	MK618599	99.93
CH12	*Microbacterium testaceum*	Citrus tree	China	MK618600	99.93
CC91	*B. licheniformis*	Citrus tree	China	MK618601	99.93
CD91	*B. methylotrophicus*	Citrus tree	China	MK618602	99.86
CE92	*B. pumilus*	Citrus tree	China	MK618603	99.93
CF91	*B. vallismortis*	Citrus tree	China	MK618604	100
CH91	*C. citreum*	Citrus tree	China	MK618605	100
CA18	*C. herbarum*	Citrus tree	China	MK618606	100
CB13	*C. luteum*	Citrus tree	China	MK618607	99.93
CD20	*C. oceanosedimentum*	Citrus tree	China	MK618608	100
CE15	*Curtobacterium* sp.	Citrus tree	China	MK618609	100
CF53	*Geobacillus stearothermophilus*	Citrus tree	China	MK618610	100
CG17	*Staphylococcus epidermidis*	Citrus tree	China	MK618611	100
CH31	*B. aryabhattai*	Citrus tree	China	MK618612	100
CA98	*B. huizhouensis*	Citrus tree	China	MK618613	99.86
CB15	*B. hunanensis*	Citrus tree	China	MK618614	99.93
CC49	*B. koreensis*	Citrus tree	China	MK618615	99.86
CD17	*B. niacini*	Citrus tree	China	MK618616	100
CE43	*B. pseudomycoides*	Citrus tree	China	MK618617	99.93
CF46	*B. stratosphericus*	Citrus tree	China	MK618618	100
CG27	*Brachybacterium* sp.	Citrus tree	China	MK618619	100
CA23	*Enhydrobacter* sp.	Citrus tree	China	MK618620	99.93
CB30	*Enterobacter* sp.	Citrus tree	China	MK618621	99.79
CC54	*Lentibacillus populi*	Citrus tree	China	MK618622	99.93
CD65	*Lysinibacillus massiliensis*	Citrus tree	China	MK618623	99.79
CE76	*Massilia* sp.	Citrus tree	China	MK618624	99.79
CF76	*Moraxella osloensis*	Citrus tree	China	MK618625	99.65
CG35	*Oceanobacillus kimchii*	Citrus tree	China	MK618626	99.93
CH87	*Paenibacillus amylolyticus*	Citrus tree	China	MK618627	99.93
CH03	*P. silvae*	Citrus tree	China	MK618628	99.86
CB34	*Pantoea eucrina*	Citrus tree	China	MK618629	99.86
CD48	*P. septica*	Citrus tree	China	MK618630	99.65
CE44	*Proteus mirabilis*	Citrus tree	China	MK618631	99.86
CG42	*Sphingobium yanoikuyae*	Citrus tree	China	MK618632	100
CH90	*Sphingomonas endophytica*	Citrus tree	China	MK618633	99.85
CA25	*S. paucimobilis*	Citrus tree	China	MK618634	99.71
CA01	*S. yunnanensis*	Citrus tree	China	MK618635	99.93
CC01	*S. saprophyticus*	Citrus tree	China	MK618636	99.93
CE01	*Staphylococcus* sp.	Citrus tree	China	MK618637	99.72
CF01	*Terribacillus* sp.	Citrus tree	China	MK618638	99.86

^*^CGMCC15726: The strain was deposited to Chinese Culture collection Bank, Beijing and this accession number was provided.

### Detection of *C*Las

All collected citrus leaves were investigated for the presence of *C*Las with *C*Las-specific primers^37^. The number of *C*Las copies was different between symptomatic and asymptomatic plants, with symptomatic plants having lower cycle threshold (CT) values and more *C*Las copies. No *C*Las was observed in healthy (uninfected) plants. After visually assessing the leaves as symptomatic or healthy/asymptomatic, the healthy/asymptomatic leaves were distinguished based on CT values and the number of *C*Las copies, with CT values >32, 25–32, and <25 (10^0^–10^1^, 10^4^, and 10^6^ *C*Las copies/g leaves) representing healthy, asymptomatic, and symptomatic states, respectively (Table 2).

**Table 2:** Level of diseased citrus plants based on *C*Las titer.

S. No	Symptoms level	CT value	*C*Las pathogen copies/gram
1	Healthy	>32	10^0^–10^1^
2	Asymptomatic	25–32	10^4^
3	Symptomatic	<25	10^6^

CLas = Candidatus Liberibacter asiaticus; CT = Cycle threshold. Pathogen copies/gram were calculated based on the standard curve of recombinant plasmid pUC18–382-HLB generated through qPCR.

### Comparison of endophytes based on disease state

**Figure 4:**
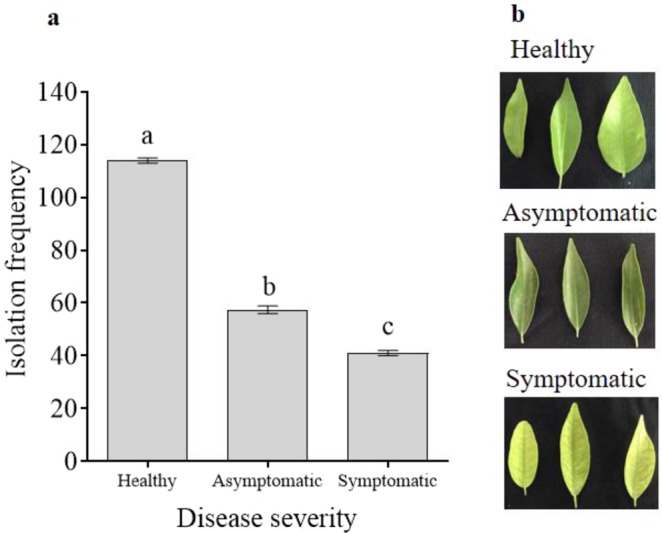
Total isolation frequency of native endophytic bacteria in healthy, symptomatic, and asymptomatic citrus trees from different provinces in China. (**a**) Total number of endophytes by huanglongbing disease state (confirmed using qPCR targeting the ribosomal protein L12 [rplL] of the *Candidatus* Liberibacter asiaticus [*C*Las] pathogen). (**b**) The disease states of the leaves were assessed before endophyte isolation from specific citrus varieties. Data were analyzed using analysis of variance (ANOVA) followed by Duncan’s multiple range test (*p* < 0.05). (**a**–**c**) Indicate significant differences among leaves with different disease states and error bars indicate the standard error of the mean (SEM).

The endophyte isolation frequency was significantly different between leaves with different disease states, with the highest numbers in the healthy leaves and the lowest in the symptomatic leaves (*p* < 0.05) (Fig. 4). Among the 114 bacterial endophytes isolated from the healthy leaves, the most dominant were *B. subtilis*, *B. velezensis*, *C. luteum*, *S. endophytica*, *B. tequilensis*, *P. amylolyticus*, and *M. testaceum*. Among the 41 endophytes isolated from the symptomatic trees, *Bacillus* sp. was the most dominant endophyte followed by *Curtobacterium*, and among the 58 endophytes isolated from the asymptomatic leaves, *Bacillus* sp. and *B. megaterium* were the most dominant endophytes. None of the other endophytes in healthy leaves were found in the symptomatic or asymptomatic leaves. Although *Bacillus* sp. was frequently isolated from all leaves, the isolation frequencies were considerably lower in the asymptomatic and symptomatic leaves compared to the healthy leaves. Most of the endophyte species were isolated at low frequencies from the various citrus varieties and regions.

### Number of endophyte species among different citrus varieties

We assessed the native endophyte communities in 24 citrus varieties. Valencia orange had the maximum number of endophyte species (22 species), while *C. reticulata* cv. Ponkan had the second highest number of endophyte species (14 species), followed by *C. reticulata* Blanco, *C. unshiu* Marcov. forma Miyagawa-wase × *C. sinensis* (L.) Osbeck, and *C. reticulata* (which all had 12 species) (Table 3).

**Table 3:** Different bacterial endophyte species from different citrus varieties and most dominant endophyte species in each citrus variety.

No’s	Citrus varieties	Endophyte species	Dominant species
1	Valencia Orange	22	*Bacillus subtilis*, *Bacillus* sp., *B. velezensis*, *B. amyloliquefaciens*, *B. megaterium*, *B. tequilensis*, *Curtobacterium luteum*, *Gamma proteobacterium symbiont of Plautia stali, Sphingobium yanoikuyae*, *S. endophytica*, *S. paucimobilis*, *S. yunnanensis*, *Staphylococcus* sp.
2	*Citrus reticulata* cv. Ponkan	14	*B. subtilis*, *Bacillus* sp., *B. velezensis, Microbacterium testaceum, B. licheniformis, B. methylotrophicus, B. pumilus*
3	*Citrus reticulata* Blanco	12	*B. subtilis*, *Bacillus* sp., *B. velezensis*, *Curtobacterium citreum*, *C. herbarum*, *C. luteum*, *C. oceanosedimentum*, *Curtobacterium* sp., *Geobacillus stearothermophilus*
4	*C. unshiu* Marcov. forma Miyagawa-wase × *C. sinensis* (L.) Osbeck	12	*B. subtilis*, *Bacillus* sp., *Staphylococcus epidermidis*, *B. aryabhattai*, *B. huizhouensis*
5	*C. reticulata*	12	*B. subtilis*, *Bacillus* sp., *B. velezensis*, *Microbacterium testaceum*
6	*C. sinensis*	11	*B. subtilis*, *Bacillus* sp., *B. licheniformis*, *B. methylotrophicus*, *B. pumilus*
7	Puzao	11	*Bacillus* sp., *B. velezensis*, *B. amyloliquefaciens*, *B. megaterium*, *B. tequilensis*, *Curtobacterium* sp.
8	Huangyan	10	*B. huizhouensis*, *B. hunanensis*, *B. koreensis*, *B. subtilis*, *Bacillus* sp.
9	*C. reticulata* (L.) Blanco cv. Nanfengmiju	10	*B. niacini*, *B. pseudomycoides B. subtilis*, *Bacillus* sp.
10	*C. maxima* cv. Sanhongmiyou	8	*B. stratosphericus*, *Brachybacterium* sp., *B. subtilis*, *Bacillus* sp.
11	Chishu	8	*C. oceanosedimentum*, *Enhydrobacter* sp., *B. subtilis*, *Bacillus* sp.
12	*C. grandis* (L.) Osbeck cv. Guanximiyou	7	*Enterobacter* sp., *B. subtilis*, *Bacillus* sp.
13	*C. reticulata* cv. Tankan	6	*Lentibacillus populi*, *Lysinibacillus massiliensis*, *B. subtilis*, *Bacillus* sp.
14	*C. unshiu* Marc	6	*B. subtilis*, *Bacillus* sp., *Massilia* sp.
15	*C. sinensis* (L.) Osbeck	4	*B. subtilis*, *Moraxella osloensis*, *Bacillus* sp.
16	Juhong orange	4	*Oceanobacillus kimchii*, *B. subtilis*, *Bacillus* sp.
17	*Fortunella margarita* (L.) Swingle	4	*Paenibacillus amylolyticus*, *B. subtilis*, *Bacillus* sp.
18	*C. limon* (L.) Burm. f.	4	*B. subtilis*, *Bacillus* sp., *P. silvae*, *Pantoea eucrina*, *P. septica*
19	Tezao	4	*Proteus mirabilis, B. subtilis*, *Bacillus* sp.
20	*C. sinensis* Osb. (navel orange)	3	*B. subtilis*, *Bacillus* sp.
21	*C. reticulata* cv. Shatangju	2	*B. subtilis*, *Bacillus* sp.
22	*C. reticulata* cv. Suavissima	2	*Terribacillus* sp., *S. saprophyticus*
23	*C. tangerina*	2	*Bacillus* sp., *B. velezensis*
24	*C. reticulata* Blanco var. Gonggan	2	*B. velezensis*, *Bacillus* sp.

### Endophyte species common to multiple citrus varieties

There were 19 endophyte species in many of the citrus varieties from each region. These endophytes were *B. subtilis, Bacillus sp., B. velezensis, B. amyloliquefaciens, B. megaterium, B. tequilensis, C. luteum, Gammaproteobacterium symbiont of Plautia stali, M. testaceum, B. licheniformis, B. methylotrophicus, B. pumilus, B. vallismortis, C. citreum, C. herbarum, C. oceanosedimentum, Curtobacterium sp., G. stearothermophilus, and S. epidermidis*. They were isolated from many of the citrus varieties, including *C. reticulata Blanco, C. sinensis* (L.) Osbeck, *C. reticulata* cv. Tankan, *C. unshiu* Marcov. forma Miyagawa-wase × *C. sinensis* (L.) Osbeck, *C. reticulata* cv. Shatangju, *C. maxima* cv. Sanhongmiyou, *C. reticulata* Blanco var. Gonggan, *C. reticulata*, *C. reticulata* cv. Suavissima, *C. grandis* (L.) Osbeck cv. Guanximiyou, *C. sinensis, C. tangerine, C. unshiu* Marc, Huangyan, Juhong orange, *C. reticulata* (L.) Blanco cv. Nanfengmiju, *Fortunella margarita* (L.) Swingle, Valencia orange, *C. limon* (L.) Burm. f., *C. reticulata* cv. Ponkan, and *C. sinensis* Osb. (navel orange).

### Endophyte species in leaves with different disease states

**Figure 5:**
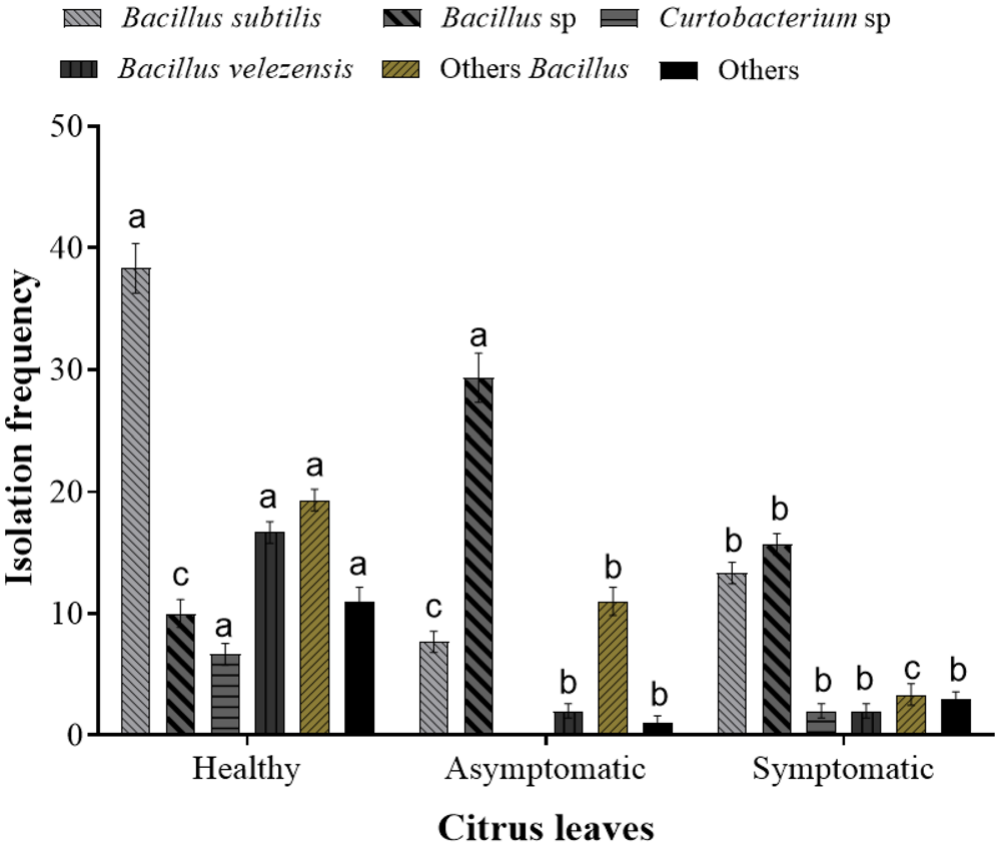
Comparison of endophyte isolation frequency by huanglongbing disease state. Means with the same letter are not significantly different based on the Tukey test (*p* < 0.05) and error bars indicate the standard error of the mean (SEM).

The dominant endophytes in different citrus varieties were compared, and *B. subtilis* was found to be the most frequent species in the healthy citrus plants and it was also recovered from a few asymptomatic and symptomatic trees (Fig. 5). *B. subtilis* showed significant differences between healthy trees and both symptomatic and asymptomatic trees (*p* < 0.05). The other dominant species across all disease states were *Bacillus* sp., *B. velezensis*, *B. amyloliquefaciens*, *B. megaterium*, *B. tequilensis*, *C. luteum*, *Gammaproteobacterium symbiont of Plautia stali*, and *M. testaceum*. In contrast, *C. luteum*, *S. endophytica*, *P. amylolyticus*, *M. osloensis*, and *P*. *septica* were frequently isolated only from healthy leaves. Interestingly, only *Bacillus* sp. was frequently recovered from asymptomatic leaves (*p* < 0.05), and it may provide resistance against *C*Las.

## Discussion

To our knowledge, this is the first study regarding the biogeographical diversity of endophytes isolated from citrus trees in most of the citrus growing regions in China. We isolated 213 endophytes from 24 citrus varieties. Healthy trees had more endophytes compared to symptomatic and asymptomatic trees, which may have been because the healthy trees were free of *C*Las. Previous studies reported that microbial communities in citrus trees are negatively affected by *C*Las^37,41,42^. In addition, microbial colonization of the branches, stems, roots, and leaves are affected by various factors. Microbial communities are present in the spatial environment inside plants, depending on their interactions inside the plants and the presence of pathogens^43,44^. Moreover, plant conditions pose a threat to the native microbial communities^36^. Similar findings using clone library and qPCR techniques were reported previously^45^. Pathogen infection of a plant drastically changes the native microbial communities and other potential beneficial microorganisms in the host. A previous study involving a clone library analysis revealed the various culturable bacteria in both *C*Las-infected and uninfected citrus roots with respect to recovery and frequency of isolated bacteria. In our analysis, only citrus leaves were used to assess the endophytic diversity, which resulted in higher proportions of *Actinobacteria* and *Firmicutes* than those reported in other studies^46,47^.

We assessed the microbial diversity in leaves from all sampled citrus varieties and the endophyte isolation frequencies were maximum. Citrus leaves (rather than branches) from sweet orange and tangerine are the preferred niche from which to isolate endophytic bacteria^48^. The endophytic bacterial population native to citrus leaves has been reported to be diverse^49^. A previous microbial diversity assessment of symptomatic and asymptomatic *C*Las-infected *Citrus sinensis* groves revealed that citrus leaves have a large core microbiome^50^. In our study, *Bacillus* and *Curtobacterium* were dominant in symptomatic plants. Previous studies also found these bacteria in symptomatic and asymptomatic citrus plants, and they had promising effects on plant growth along with biocontrol abilities^51–53^.

Although *C*Las can colonize some plants without inducing any apparent HLB symptoms, the total number of endophytes were higher in these asymptomatic trees than in symptomatic trees. In contrast, a previous study reported lower endophytic diversity in *Xylella fastidiosa-*infected asymptomatic citrus plants compared to *Xylella fastidiosa-*infected symptomatic plants due to *X. fastidiosa* resistance in the former^48^. The *Bacillus* sp., *Curtobacterium* sp., *Enterobacter* sp., and *Pantoea* sp. found in our study were also reported in sweet orange and tangerine infected with *X. fastidiosa* (which causes citrus variegated chlorosis) in Brazil^36^, but the species in our study were different from those reported in HLB-infected citrus trees in Florida^50^. The differences could be due to differences in tissue samples (leaf, midrib, or branch), different environmental conditions (such as the weather), and the dominant HLB pathogen in each geographical area. We found that the dominant endophytic genus in most of the citrus varieties was *Bacillus* (few of these bacteria can fix nitrogen^54,55^, and they can colonize a diverse range of plants^36,48^), and higher *Bacillus* density was associated with lower HLB severity (from healthy to asymptomatic and finally to symptomatic leaves). Endophyte colonization of citrus plants may depend on the HLB disease state (related to the *C*Las strain), with a potential synergistic interaction between endophytic *Bacillus* and *C*Las in order to mitigate HLB. However, as *C*Las is non-culturable in axenic cultures, we did not explore the interactions between the endophytes and *C*Las.

Several interesting bacteria were recovered from asymptomatic and healthy plants, indicating their potential association with HLB resistance. Another important endophyte recovered from healthy and symptomatic plants was *Curtobacterium*, which has an important role against several plant pathogens due to antimicrobial production and its ability to induce systemic resistance^56,57^. *Curtobacterium* may be useful for limiting the *C*Las infection of symptomatic plants, but the mechanism needs further exploration. *Bacillus* and *Curtobacterium* in the inner parts of healthy and asymptomatic plants may enhance HLB resistance by producing antimicrobials or triggering a degree of *C*Las resistance. The frequent recovery of *Bacillus* and *Curtobacterium* from healthy and asymptomatic plants supported our hypothesis regarding possible colonization of the citrus plants with these endophytes.

Furthermore, we tested various citrus varieties to find the one with the largest number of endophyte species and to identify the endophyte species that were common among many citrus varieties. Valencia orange (*C. sinensis*) had the maximum number of endophyte species (22 species), followed by *C. reticulata* cv. Ponkan (14 species). In addition, 19 endophytes species were observed in most of the citrus varieties; the most dominant were *B. subtilis*, *Bacillus* sp., *B. velezensis*, and *B. amyloliquefaciens*. The most dominant endophytes in various citrus varieties were *Bacillus* sp., *B. velezensis*, *B. amyloliquefaciens*, *B. megaterium*, *B. tequilensis*, *C. luteum*, *Gammaproteobacterium symbiont of Plautia stali*, and *M. testaceum*. These dominant bacteria were also observed previously in maize^58^ and citrus^59^. In contrast, *C. luteum*, *S. endophytica*, *P. amylolyticus*, *M. osloensis*, and *P*. *septica* were frequently isolated only from healthy citrus leaves. Several factors including environmental conditions, inoculum density, host developmental stage, and host species influence the endophytes found in specific plants^60^. A previous study also recovered *Microbacterium, Lysinibacillus, Brevibacillus*, and *Variovorax* more frequently^46^. We found that the total number of endophytes isolated from most of the citrus varieties was 10^4^–10^6^ CFU/g of leaves. Endophyte colonization is regulated by factors such as plant growth stage, environmental conditions, seasonal variation, plant cultivar and, most importantly, plant genotype^61,62^. The role of specific endophytic communities in nature is an important clue to select endophytes with potential beneficial bioactivity.

Taking the results together, this is the first large-scale study in China showing diverse bacterial endophytic communities in various citrus varieties from nine citrus growing regions. The diversity of the citrus microbiomes in trees from different geographical areas was assessed. *Bacillus subtilis* and *Bacillus* sp. was the dominant genus in healthy and asymptomatic trees, respectively. The number of endophytes depended in each region on the number of citrus varieties. Moreover, the endophytic communities in healthy, asymptomatic, and symptomatic leaves varied, as reported previously^41^, revealed interactions among pathogenic and beneficial microbial communities inside citrus plants. The functional influences of endophytic communities on citrus plants need to be explored. In the long run, specific beneficial microbiomes from citrus trees may have a role in citrus growth promotion and combating HLB and other pathogens.

### Originality significance statement

The authors confirm that all the reported work is original and, to our knowledge, this is the first report on the endophytic community diversity in citrus trees in nine citrus growing regions in China. The results indicate that huanglongbing disease negatively affects the native endophytes because the healthy trees had more endophytes than the symptomatic and asymptomatic trees. We could potentially use endophytes to combat huanglongbing disease in the future.

## Supplementary information

Figure S1, Figure S2, Figure S3, Table S1, Figure S4.
