# Supplementary material for: Core endophyte communities of different citrus varieties from citrus growing regions in China
Source: Sci Rep. 2020 Feb 27;10:3648. doi: 10.1038/s41598-020-60350-6 (PMC7046616; doi:10.1038/s41598-020-60350-6)
Supplement: Supplementary file 1 — Figure S1, Figure S2, Figure S3, Table S1, Figure S4. [file 41598_2020_60350_MOESM1_ESM.docx]

**Core endophyte communities of different citrus varieties from citrus growing regions in China**

Shahzad Munir^1^, Yongmei Li^1^, Pengfei He^1^, Huang Min^2^, Pengbo He^1^, Pengjie He^1^, Wenyan Cui^1^, Yixin Wu^3,4^, Yueqiu He^1,3,4^

^1^State Key Laboratory for Conservation and Utilization of Bio-resources in Yunnan, Yunnan Agricultural University, Kunming 650201, Yunnan, China

^2^Agriculture College and Urban Modern Agriculture Engineering Research Center, Kunming University, Kunming 650214, Yunnan, China

^3^National and Local Joint Engineering Research Center for Screening and Application of Microbial Strains, Kunming 650217, Yunnan, China

^4^ Faculty of Agronomy and Biotechnology, Yunnan Agricultural University, Kunming 650201, Yunnan, China

*** Corresponding author:**

State Key Laboratory for Conservation and Utilization of Bio-resources in Yunnan, Yunnan Agricultural University, Kunming 650201, Yunnan, China

Email: [ynfh2007@163.com](mailto:ynfh2007@163.com)

**Running title:** Microbial communities in citrus growing regions in China

**Originality Significance Statement**

The authors confirm that all the reported work is original and, to our knowledge, this is the first report on the endophytic community diversity in citrus trees in nine citrus growing regions in China. The results indicate that huanglongbing disease negatively affects the native endophytes because the healthy trees had more endophytes than the symptomatic and asymptomatic trees. We could potentially use endophytes to combat huanglongbing disease in the future.

| 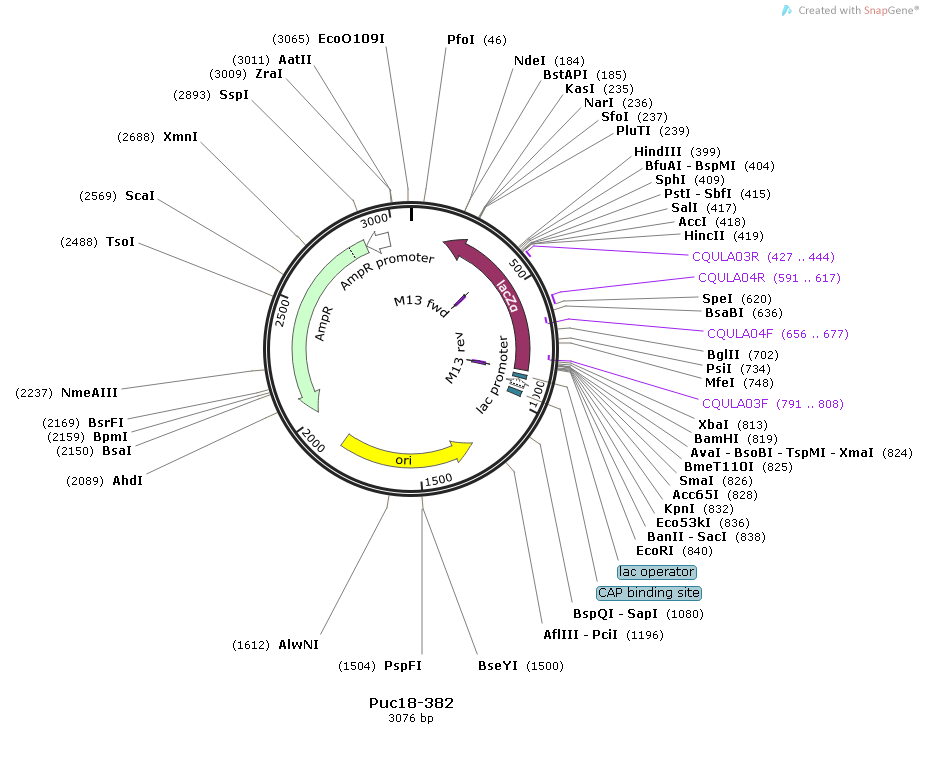  **Fig. S1** Map of recombinant plasmid pUC18-382-HLB. |
| --- |

| 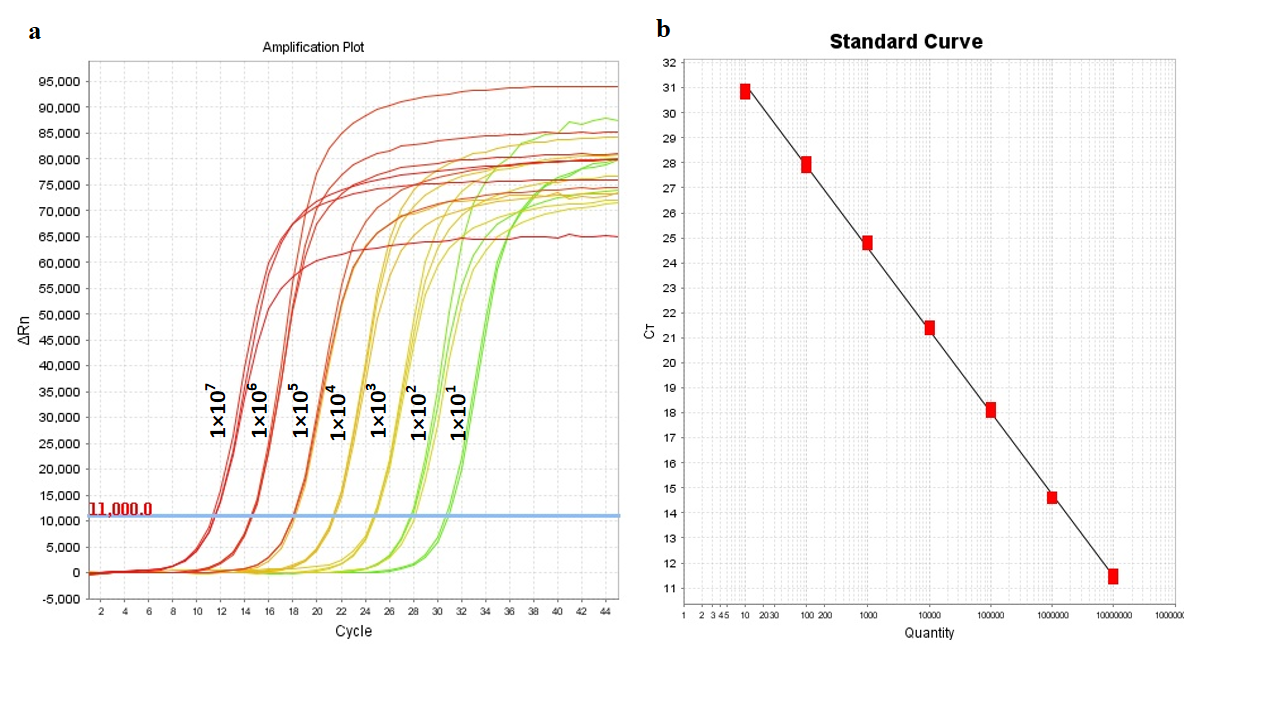  **Fig. S2** (a) Amplification plot for recombinant plasmid pUC18-32-HLB. (b) 10-fold dilutions of the recombinant plasmid were used to generate a standard curve. The CT values ranged from 11.473 to 30.841. The linear equation generated using the standard curve was CT = -3.275×pathogen copies + 33.934, which was used to calculate the number of *Candidatus* Liberibacter asiaticus (*C*Las) pathogen copies/g of leaves. |
| --- |

| ^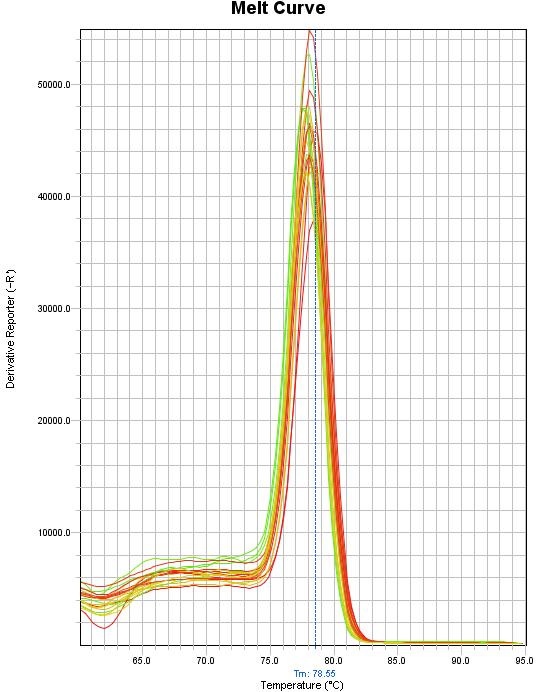^  **Fig. S3** Melting curve of SYBR Green assay for Ca. Liberibacter asiaticus using dilutions of recombinant plasmid pUC18-382-HLB using Real Time PCR (Applied Biosystems). The peak at 78°C confirms the successful amplification. |
| --- |

| **Table S1** Total culturable bacterial endophytes from all the citrus cultivars from 9 provinces of China | | |
| --- | --- | --- |
| **S.No** | **Citrus cultivars** | **Total culturable endophytes (CFU/gram)** |
| 1 | *Citrus reticulata*Blanco | 8.35×10^5^ |
| 2 | *C. sinensis* (L.) Osbeck | 8.82×10^6^ |
| 3 | *C. reticulata* cv. Tankan | 7.46×10^5^ |
| 4 | *C. unshiu* Marcov. forma Miyagawa-wase*× C. sinensis* Osbeck*)* | 2.40×10^5^ |
| 5 | *C. reticulata* cv. Shatangju | 3.59×10^5^ |
| 6 | *C. maxima* cv. Sanhongmiyou | 3.50×10^5^ |
| 7 | *C. reticulata* Blanco v. Gonggan | 4.00×10^5^ |
| 8 | *C. reticulata* | 6.40×10^5^ |
| 9 | *C. reticulata* cv. Suavissima | 6.42×10^6^ |
| 10 | *C. grandis* (L.) Osbeck cv. Guanximiyou | 6.73×10^5^ |
| 11 | *C. sinensis* | 4.59×10^5^ |
| 12 | *C. tangerina* | 6.97×10^6^ |
| 13 | *C. unshiu* Marc | 3.58×10^6^ |
| 14 | Huangyan | 5.00×10^5^ |
| 15 | Juhong orange | 2.87×10^5^ |
| 16 | *C. reticulata (*L.)Blanco cv. Nanfengmiju | 2.62×10^5^ |
| 17 | *Fortunella margarita* (L.) Swingle | 1.32×10^5^ |
| 18 | *Valencia Orange* | 2.14×10^4^ |
| 19 | *C. limon*(L.) Burm. f. | 2.00×10^4^ |
| 20 | *C. reticulata* cv. Ponkan | 1.67×10^4^ |
| 21 | *C. sinensis*Osb*.* (Naval orange) | 8.67×10^4^ |
| 22 | Puzao | 9.64×10^4^ |
| 23 | Tezao | 1.71×10^5^ |
| 24 | Chishu | 8.20×10^4^ |

**
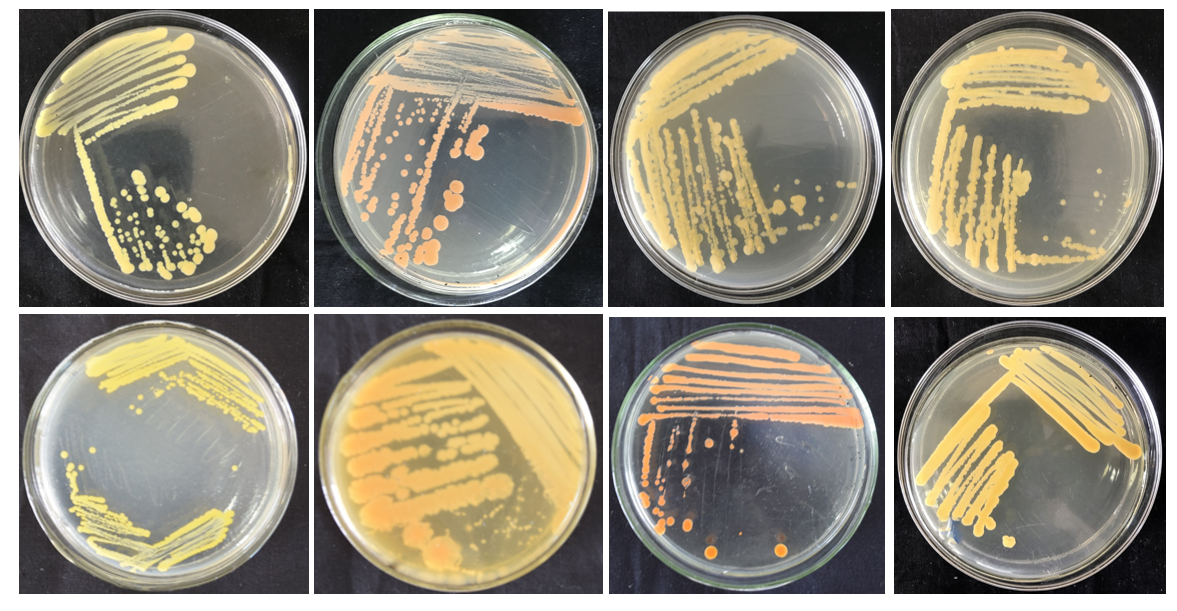
**

**Fig. S4** Culturable bacterial endophytes after successful purification on LB medium after 48-72 hours of culturing.
